# It Takes Time to Unravel the Ecology of War in Gaza, Palestine: Long-Term Changes in Maternal, Newborn and Toddlers’ Heavy Metal Loads, and Infant and Toddler Developmental Milestones in the Aftermath of the 2014 Military Attacks

**DOI:** 10.3390/ijerph17186698

**Published:** 2020-09-14

**Authors:** Nabil al Baraquoni, Samir R. Qouta, Mervi Vänskä, Safwat Y. Diab, Raija-Leena Punamäki, Paola Manduca

**Affiliations:** 1 Faculty of Medicine, Islamic University of Gaza, Gaza, Palestine; nbarqouni@gmail.com; 2 Doha Institute for Graduate Studies, School of Social Sciences and Humanities, Al Tarfa Street, Zone 70, Doha, P.O. Box 200592, Qatar; samir.qouta@dohainstitute.edu.qa; 3 Department of Psychology, Faculty of Social Sciences, Tampere University, 33014 Tampere, Finland; mervi.vanska@tuni.fi (M.V.); safwatdiab@hotmail.com (S.Y.D.); raija-leena.punamaki-gitai@tuni.fi (R.-L.P.); 4 Association for Scientific Research, Nwrg-onlus, 16123 Genova, Italy

**Keywords:** heavy metal war remnant, metal load in infant and in utero, developmental milestones, child growth restriction

## Abstract

Toxicant, teratogen and carcinogen metal war remnants negatively affect human health. The current study analyzes, first, the persistence of heavy metal contamination in newborn hair in four cohorts across time in Gaza Palestine; second, the change in mothers’ and infants’ heavy metal contamination from birth to toddlerhood; and third, the impact of heavy metal contamination on infants’ and toddlers’ growth and development. The hair of newborns was analyzed for twelve heavy metals by Inductively Coupled Plasma Mass Spectrometry (ICP/MS) in cohorts recruited at delivery in 2011, 2015, 2016, and 2018–2019. In the 2015 cohort, mothers’ hair samples were taken at delivery, and toddlers and mothers hair were also analyzed 18 months later. Growth levels of infants at six months and toddlers at 18 months were assessed according to World Health Organization (WHO) standards according to a mother report and pediatric check-up, respectively. 1. The level of metal contamination in utero was persistently high across 8 years, 2011, 2015, 2016, 2019, following three major military attacks (2009, 2012, 2014). 2. The 2015 cohort babies exposed in utero to attacks in 2014 at six months showed association of high load at birth in mother of arsenic and in newborn of barium with underweight, of barium and molybdenum in newborn with stunting. 3. Eighteen months after birth, toddlers had a higher level of metals in hairs than when they were born, while, in their mothers, such levels were similar to those at delivery, confirming persistence in the environment of war remnants. Underweight and stunting, both in infants and toddlers, were higher than reported for previous years, as well as being progressive within the cohort. Severe environmental factors, metal contamination and food insecurity put Gaza’s infant health at risk.

## 1. Introduction

New-generation weapons, used in current wars and military operations, expose civilians to severe health hazards through heavy metal contamination [1]. Pregnant women and their progeny are particularly vulnerable to heavy metal intake, affecting mother metabolic changes and fetal development [2]. Studies show the negative impact of maternal prenatal exposure to heavy metal war remnants on newborn health, including increased rates of birth defects and preterm babies [3,4,5,6,7,8]. Meanwhile, very little has been reported about the long-term impact of mother and fetus contamination on the health and development of infants and toddlers.

In Gaza, studies in different cohorts of pregnant women reported high levels of heavy metal contaminants since 2011 (after the 2009 military attacks), and continuing for at least 5 years after the further attacks of 2012 and 2014 [6,8], but the level of contamination acquired in utero by the progeny in these chronically exposed mothers remained undocumented.

Heavy metals are classified according to their effects: toxicants (e.g., barium, aluminium) can cause acute poisoning, carcinogens (e.g., arsenic, cadmium) induce cancers and congenital malformations, neurotoxins (e.g., lead and manganese) are particularly harmful to the brain, and teratogens (e.g., mercury, uranium) to the fetus development [9,10]. Finally, microelements (e.g., selenium) are necessary for most living organisms in small quantities, but both their deficiency and over-dose form risks to health. The mechanism of biological action of most metal is epigenetic [11,12].

People living in warzones become exposed to heavy metals delivered by weapons [1,8] through contamination at their explosion [13] and, due to metals resilience in the environment, they are exposed for long term to weapons-remnants in/on the ground. The population can assume metals from the environment via air, food, water, and skin contact.

Military metals detected in Gaza include those in penetrator bombs heads and ammunitions, metal components of white phosphorus shells, and weapons enhanced through utilization of heavy metals as augmenters or as primary effective agents of combustion and blast. Remnants of these weapons have been found to contain numerous toxic, teratogen and carcinogen heavy metals [1] and were found in Gaza in hair of children after the Israeli military operation in 2009 [14].

Exposed pregnant women transfer the contamination to the fetus, through the placenta [15,16]. The rapid growth and development throughout pregnancy makes the fetus particularly sensitive to external influences, such as contamination by heavy metals which causes long-term risk for health at birth and can interfere with infant and child development. Expert reports from Iraq and Gaza, Palestine, show a sudden increase in the prevalence of malformations at birth and in babies born preterm during in the years following USA or Israeli military attacks, particularly high in the areas most severely shelled by modern weaponry [3,4,5,6,7,8,17]. Mothers and newborns living in Gaza, Palestine, after the 2014 attacks by Israel on Gaza were found to have increased toxic and teratogen metal loads compared to international standards of reference from a non-war area, with loads in newborns correlating positively with maternal ones [18]. Newborns of Gaza mothers, who were directly exposed to military attacks during the 2008/09 attacks on Gaza, were at increased risk of prematurity and birth defects, and the newborns with birth defects or preterm had a higher level of mercury barium and tin, as compared to normal newborns [6].

Fetal programming refers to the ability of environmental factors during the prenatal development to adjust relevant physiological parameters in the fetus [2]. Importantly, the adjustments can endure into adulthood, producing non-genetic disorders or dysfunctions and even affect the next generation [19,20,21,22,23,24,25,26,27,28,29,30,31,32,33,34,35,36].

The main mechanisms through which prenatal heavy metal exposure may alter children’s health and development relate to epigenetic, hormonal and nutritional factors. First, there is evidence of heavy metals inducing epigenetic modifications in the maternal, fetal and placental genome [19,20,21,22,23,24,25]. This can happen through changes in the methylation of DNA, histone modifications, and the expression of noncoding RNAs that in turn may permanently affect both the mother and the child [24]. Second, heavy metals can cross the placenta, resulting with their concentrations in the umbilical cord blood being for some of them for instance lead, mercury and cadmium, even markedly higher than in the maternal blood, with potential long-term adverse effects on the child [26]. Another important buffering mechanism, the blood–brain barrier, is not yet fully working in the fetal period, which makes the brain particularly vulnerable to prenatal heavy metal contamination [24,25]. Third, prenatal exposure to heavy metals may disrupt vital endocrine functions. For example, metals can mimic endogen hormone activity and reproduce equivalent effects, or they can act as hormone agonists for specific receptors [28,29]. Through these mechanisms, metals can interfere mainly with steroid and thyroid hormone functioning, leading to long-term adverse effects on child growth and development. Fourth, in some studies, heavy metals have been shown to interfere with the optimal transfer of micronutrients to the fetus, e.g., the interference of cadmium with zinc transport to the fetus [30]. Finally, a reason for severe consequences of maternal prenatal toxic exposure to child development lies per se in the multitude of these risk mechanisms and, in the case of war remnants, in the complex spectrum of the contamination. Moreover, metals can accumulate in all livings, and persistent contamination poses longer-term health risks for the whole population and additional risks for the effects that it can have upon exposure in utero during the morphogenesis and the functional development of the organs in the fetus.

Although knowledge is scarce on prenatal contamination in the context of war, reviews on the impact of industrial and pollution metals confirm that that maternal prenatal exposure to heavy metals predict problems in the course and outcome of pregnancy [26,27,28,29,30,31,32,33,34,35,36,37,38,39,40,41,42,43,44]. In particular, lead, cadmium and mercury have been found to predispose pregnancies to complications, such as placental insufficiency [31,32], pre-eclampsia [32], fetal growth restriction [33,35], and preterm delivery [36,39]. In line with this, newborns exposed to prenatal maternal contamination by arsenic and cadmium are at increased risk of low birthweight and to cadmium to being small-for-gestational age [40]; arsenic and cadmium in utero exposure was associated with telomere shortening, even at a low concentration [41]; barium was associated to preterm birth and mercury to birth defects in humans [5,37]. Prenatal exposure to uranium was associated with preterm birth [42,43] and to vanadium with increased risk of adverse birth outcomes [44]. Prenatal exposure to multiple metals was associated with decreased reproductive health and negative outcome at birth [3,4,5,6,7,8].

Although lifelong implications of developmental exposures are coming to be the focus of attention, [45], research is still scarce and discrepant on impacts of maternal prenatal heavy metal contamination on later infant and toddler health and growth [46,47,48]. We could detect three studies with substantial sample sizes to support the expectation of prenatal exposure to heavy metals being harmful for children’s health and growth.

In a study of 289 mother–child dyads in Taiwan [46], found that high cord blood cadmium level predicted decreased child height, weight and head circumference growth up to three years of age. A Polish study of 379 dyads found that a high level of mercury in cord blood was associated with restricted growth of height in infancy, although not yet evident at birth [47]. A study followed over 2000 Bangladeshi mother–child dyads [48] from pregnancy to toddlerhood, and found that a high level of prenatal (urine) arsenic predicted problems in infants attaining weight and height from three to 24 months, although the association was markedly attenuated after adjustment for child sex, maternal body mass index (BMI) and family socioeconomic status. Importantly, not all studies have found an impact of prenatal heavy metal exposure on later child health and growth.

Heavy metals co-occurring in human blood may increase or neutralize each other’s impact [49,50,51,52]. Therefore, studies on impacts of multiple heavy metals on newborn, infant and child health are warranted. A Chinese study of 1652 mother–infant dyads [51] examined the impact of prenatal exposure to five heavy metals (lead, mercury, cadmium, arsenic and thallium) on newborn neurobehavioral development, and found exposure to heavy metals, especially arsenic, to decrease newborn neurodevelopmental attainments. Among North American mother–toddler dyads (*N* = 92), the combination of seven heavy metals (cadmium, chromium, cobalt, lead, mercury, nickel and silver) in pregnancy predicted a high number of child infections and other illnesses at 3 years [52]. In none of these studies was the level of contamination of toddlers during their life investigated.

Contextual information: Gaza was under economic and military blockade by Israel since 2007, which hinders the import of building material and explains why debris of massive destruction and of toxic war remnants of shelling and bombardments stay permanently in the ground. The women in the cohorts studied had all lived through multiple war attacks, including three major ones in 2008–2009, 2012 and 2014, plus a myriad of more localized hits throughout. This implied a remodeling of the territory, due to extensive destruction, removal of ruins and rebuilding, and also major internal displacement. Each of these events produced a movement on the ground of stable war remnants as heavy metals. Major block of local productions, decline in use of gasoline for energy production and of road traffic, increased food dependence from international aid, were also prominent features of the last 13 years [53,54].

Heavy metals deriving from new generation weapons co-occur. Wound tissues of war injuries in Gaza were found to contain numerous heavy metals with toxicant, teratogenic and carcinogenic effects on human body [1] and these were detected in 60% of the 96 children hair tested one year after the military attacks [14] and in women exposed to attacks 6–9 months earlier [18]. The acute contamination to which pregnant women and the fetus were exposed at the time of attacks [18], as reported in other studies [4,8,55], was indeed followed by chronic exposure of the mothers to almost unabated levels of metals persisting in the environment in Gaza.

The present study aims, first, to compare the persistence of metals contamination in the hair of four newborn cohorts: after 2008–09 Israeli attacks (2011 cohort), after the 2012 and 2014 major attacks on Gaza (2015 and 2016 and 2018–2019 cohorts).

Second, we investigate the relevance of metal contamination in utero on the growth (weight, height, head circumference) and health measurable classified according to the WHO [56], at 6 and 18 months of age of the cohort of babies born in 2015 to mothers who were at their first trimester of pregnancy during the attacks in 2014.

Third, we analyze the patterns of contamination in infants of the 2015 cohort when they reach 18 months of age, and retest for contaminants their mothers, to assess the kind and amounts of war remnants still bioavailable for uptake by people, as a measure of ongoing environmental contamination.

Fourth, we examine if the level of metals assumed from the environment by toddlers is correlated with any of their pediatric outcomes.

## 2. Methods

### 2.1. Study Participants

In this study, we investigate the level of metal contamination attained during fetal development in different cohorts of babies, born in 2011, 2015, 2016, 2018–2019.

In the cohort of babies born in 2015, we study the association with health measurables at 6 months of age of the babies of the load of metals detected at birth in their hair and in that of their mothers.

When the infants become toddlers of 18 months, we analyze the load of contaminants in their hair as a measure of their environmental exposure and discuss it in comparison to their contamination as newborns; we further examine how maternal and foetal prenatal heavy metal contamination predicts toddler development and if the contamination of toddlers is correlated with their pediatric milestones.

The participants for the study at birth in the years 2011, 2016, 2018–2019 were Palestinian women recruited at their delivery in Al Shifa hospital maternity units, Gaza city, respectively, 4049 women in 2011, 6104 women in 2016, 4767 women in 2018–2019. In addition, 502 women were recruited in 2015 (January-March) at maternity units in four hospitals from 4 governorates in the Gaza Strip (al Shifa, Gaza; al Taheer, Kan Younes; Al Aqsa, Middle area; Al Awda, North). All women registered at birth delivered live babies at 28 or more gestational weeks. The inclusion of mothers was in sequence of deliveries for 2011, 2016 and 2018–2019, and during one shift/day for those in 2015. These last women had been pregnant in their first trimester during the 2014 military attacks on Gaza, 55 days long. All women in all cohorts gave their voluntary consent to participating in the study and there were no refusals. The midwifes collected data at birth and maternal and newborn hair samples. Diagnoses were corroborated by the neonatologist of the hospitals (Dr. N.Bobol and Dr. K. Bawab) under the supervision of Dr. N.Al B. and Prof. P.M.).

Only 392 women among the 502 enlisted at delivery in 2015 (here referred as T = 1), i.e., 78%, could be reached when their infants were 6–7 months old (*M* = 6.2 *SD* = 0.4), here referred to as T = 2, and were visited in their homes. Missing results were due to a transfer of residence or a change of phone number.

The 392 women that were included at T2 were further invited for a pediatric standard health assessment of the child at 18 months of age (T = 3) and 308 of them reported, i.e., 61.3% of the original cohort. No motivation was given for not participating, but problems with transport or household arrangements are the likely reasons, as the visits took place in the doctor’s office.

### 2.2. Measures

Registration and sample collection at birth: Protocol for registration at birth was the same, with minor variations, for all years of study. It was inclusive of standard European and US birth register information and in addition included questions about environmental exposures, as reported in detail before [4,5,6,18]. It included child’s health by CDC10, Apgar score, birthweight, birth order, and need of intensive care unit (NICU), mother’s health, length of gestation, mode of delivery, education, nutritional and socio economic situation of the family, parents familiarity, and health issues in the extended kin. The aim of the data collection coincides with WHO stated goal of defining the environmental determinants of reproductive health, and we developed “ad hoc” questionnaires for the registration of births integrating the standard EUROCAT questions [57]; more than 80 queries accounting for the peculiar conditions due to the blockade of Gaza and to the local habits were included, such as questions about exposures to attacks and war remnants. The last were prepared by taking into account reports of war damages and of weaponry used by the UN mission for documentation and retrieval of spent weaponry, by United Nation Development program, and of the opinion of environmental experts. The residence of the women in the cohorts was in Gaza and North governorate (respectively, 66% in 2011, 72% in 2016 and 2017 and 69% in 2018–2019 resident in Gaza) and 40% in Gaza, 20% in the north, 20% in the south and 20% in the middle governorates in 2015.

### 2.3. Data Collection at T2

Data collection at T2 was conducted by ten fieldworkers with BA degrees in a relevant field and with former experience in research work, after comprehensive training on the research tasks and procedures. The interviews of 392 mothers took place in the participants’ homes or in the governmental Primary Health Care Centres (PHCC) in June–October 2015, when the infants were 6–7 months old (*M* = 6.2, *SD* = 0.4). Two members of the research team (S.D. and S.Q.) supervised the fieldwork. The drop out between T1 and T2 was 22%. Reasons for dropping out from the study were the death of the baby (*n* = 12), family reasons, and incorrect or changed home address after displacement resulted from the war. The families that dropped out did not differ from those who remained in terms of child’s gender or birthweight, or the age and education of the mother and the father.

### 2.4. Data Collection at T3

The infants were, on average, 18–19 months (*M* = 18.0, *SD* = 1.35) old in August–December 2016, when 308 of them were visited and their health scores were registered by one of 5 pediatricians, in this study, in each of the areas of residence of the family (Supervisor Dr. N. Al B.). The medical report was prepared according to WHO protocol and standards [56]; this is under the assumption that “The new standards demonstrate for the first time ever that children born in different regions of the world, when given the optimum start in life, have the potential to grow and develop to within the same range of height and weight for their age”.

The developmental milestones covered weight (grams), height (cm), weight per height, head circumference (cm), and muscular development (standing and moving), indicating neurological and sensorimotor development for age. The assessments were scores as below average (2SD), average, and above (2SD) average based on the growth charts for girls and boys from birth to 36 months. Hair samples of the mother and the infant were collected by the pediatrician.

### 2.5. Hair Collection for Determination of Metal Load

The load of metals was measured using Inductively Coupled Plasma Mass Spectrometry (ICP/MS) [58] in mother and newborn hair samples collected at birth and for the cohort of the year 2015 at 18 months after birth.

The number of samples for which hair metal load was determined is indicated in the tables and it was a random sample for the years 2011, 2016, 2018–2019 and was for all 502 cases registered at birth in 2015. Of these, only a randomly chosen subgroup was analyzed also at 18 months of age of the infant, as indicated in the tables. Heavy metal concentrations are expressed in ppm (parts per million, i.e., micrograms per gram). Mother hair samples were taken from four cm nearest to the scalp at the nape of the neck and reflect environmental exposure in the last 4–5 months of pregnancy and eventual release of metals previously accumulated in-body. Hair from the newborn reflects the accumulation of metals through life in utero. Hair collected at birth from 69 newborns in 2011, 503 newborns and 502 mothers in 2015, 50 newborns in 2016 and 18 in 2018–2019 were analyzed. Hair collected from 69 infants and 79 mothers that were already tested at the time of delivery (T0) was collected 18 months later (T3) and analyzed. Hair processing and analysis was previously described [6,55]. It followed the recommendations by International Atomic Energy Agency (IAEA) 58] and was done in Pacific Rim Laboratory, ISO/Tec 17,250 accredited (Canada). The load was measured for 23 metals, including microelements and known components of weapons. Here, we report the result for 12 of the heavy metals, considered the most relevant on the basis of the high level, by comparison with a wider cohort of international standards from the outside war area [59] and, but for lead, with a smaller cohort of Italian and Israeli women, of the fact that these metals were previously identified to be weapon-delivered in Gaza [1], many of which are teratogenic or carcinogenic, and of their association with negative health at birth in Gaza [5,8]. In brief, 0.2 g of washed hair was added with 2 mL HNO3 and 2 mL H_2_O_2_, heated at 85 °C for 2 h, added at r.t. with 6 mL of water. Samples were run 3 times using Agilent 7700. The limits of detection (LOD) in ppm were: Titanium (Ti) 0.04; Barium (Ba), Cobalt (Co) and Chromium (Cr) 0.02; Arsenic (As) and Molybdenum (Mo) 0.001; Cadmium (Cd) and Uranium (U) 0.0001; Mercury (Hg) 0.0004; Selenium (Se) 0.22; Strontium (Sr) 0.01; Vanadium (V) 0.002. Experimental values below LOD for each metal were considered equal to 0.

### 2.6. Statistical Analyses

Percentage distributions were applied to illustrate the sample demographic and obstetric characteristics. The non-parametric Kruskal–Wallis tests for median differences were used to compare the newborn and infant heavy metal contamination in different time points of 2011, 2015, 2016 and 2018-19. Sign tests were used to detect the changes in the medians of the heavy metal contamination between mothers at delivery and when the infant was 18 months, and between newborns and when they were 18 months in the 2015 cohort. (The sign test is designed for pairwise comparisons, i.e., medians within the same participants assessed at these two time points.) Kruskal–Wallis tests for medians were used to answer the question of maternal and infant heavy metal contamination at delivery/birth (T0) and at 18 months (T3) associating with paediatrics’ evaluation of developmental milestones of infants (indicated by standard evaluations of growth, neurological and sensorimotor assessments for age). Concerning developmental milestones, we compared the medians of the load of heavy metals (in mothers and newborns) between the categories of below (2SD), average or above (2SD) standard values for age. The Dunn–Bronferroni post-hoc tests specified the significant differences between below vs. average vs. average vs. above and below-average. The median comparisons were chosen as metal load distributions may differ and median statistics can succinctly depict these variations. The non-parametric methods were chosen due to the non-normal distribution of experimental values. For the distribution of the differences in the median values of the hair metal concentration between 4 cohorts of newborns (at 2011, 2015, 2016 and 2018–2019), the sample sizes vary between 18–502, and, as all classes exceeded the minimum of 15, the cohort group size is estimated to be sufficient. Similarly, for the comparison of metal loads between T1 and T3 in mothers and newborn/toddlers.

For distribution of the differences in the median values of hair metal loads in mother and newborn between three classes of infant weight and length at 6 months, the sizes of the sample of participants (T2; N = 392) sufficient to detect main effects (with a power of 0.8 and a significance level of 0.05), which can be expected in developmental toxic research. Similarly, for the distribution of the differences in the median values of hair metal loads in mother and newborn at 18 months between the classes of pediatric outcomes.

## 3. Results

### 3.1. Load of Metals Assumed in Utero and Found in Newborn Hair at Various Times after Military Attacks

Two years after military attacks in the winter 2009 and from 1 to 5 years after the attacks in 2014—i.e., in 2015, 2016 and 2018–2019—the hair of newborns was collected at birth and analyzed for the load of metals with potential relevance as teratogens and/or carcinogens or essential microelements (Table 1). Samples were randomly chosen among the thousand newborns registered: N 69 for 2011, N 503 in 2015, N 50 in 2016, N 18 in 2018–2019. The registration data at birth were previously published for the cohorts in 2011, 2016 and 2018–2019, and those for the 2015 are here presented in Table S1 Supplementary.

Analytical tests show significant differences in the loads of all metals, except mercury (Hg), across the compared time points. Since 2011, the load of barium (Ba), arsenic (As) and cadmium (Cd), and, since 2015 (the first time it was tested), that of strontium (Sr), decreased in the hair of newborns; the load of uranium (U) was highest in 2011 but remained steady afterwards; vanadium (V), steady since 2011, increased in 2018–2019. Selenium (Se) was higher at all time points compared to 2011; cobalt (Co) decreased by 2016; molybdenum (Mo) and titanium (Ti) were not detected in 2011, and, for them and chrome (Cr), a clear direction of change in time was not discernible. Comparison of the loads since 2016 to 2019 (not shown) showed less differences than when 2011 was included.

### 3.2. Physical Growth of the Infants at Six Months and Association with the Load of Metals Measured in Mothers and Progeny at Delivery

Out of the 502 mothers and babies born from mothers who were in the first 3 months of their pregnancy during the attacks in 2014, six months later 392 participated to the growth assessment: 12 babies had died for unknown reasons and 22% of mothers chose not to continue their participation or were displaced to unknown address. The six months old infants were exclusively breast fed (18%), or breast fed with addition of bottled milk (72%) or only bottle fed (10%), and solid food was additionally introduced after the 5th month of life in 60% of the cases; no infant moved autonomously on the ground, and their contact with the environment was mostly mediated through milk and breathing.

The measures at T2 for weight and length of the infants were reported by mothers to the interviewers and were classified according to WHO Child Growth Standards per age (2006). Prevalence of not normative measures was the same for the two sexes: 20.1 %, (*n* = 77) infants were <2SD standard for weight, i.e., underweight, and only 1.5% (*n* = 5) > 2SD standard; 30.1%, (*n* = 102) was <2SD standard, i.e., stunting, and 6.5% (*n* = 22) > 2SD standard for height.

In Table 2, we show the significant difference found among categories for growth (height/age or weight/age) of the infant with the median metal contamination found in the hair of their mother at delivery or in that in the newborns (T1). The metals found at birth and for which there was significant association of higher amounts with impaired growth of infants were few, and not the same in mothers or newborn; higher mother load in arsenic and higher load in barium of the newborn were associated with lower weight/age of the infants; higher load of the newborn in barium and molybdenum were associated with shorter height/age. These significant inverse correlations suggest that the highest load of these metals during pregnancy might negatively affect the development of the infant at six months. Yet, as shown in Table 2, there were also correlations of high load of metals in mother or in newborn hair with infant above the WHO standard; these were five individuals overweight associated with higher load in newborns of arsenic, chrome, strontium, titanium, and 22 higher/age associated with higher load in mothers of cobalt, chrome, vanadium and in newborn of arsenic, chrome, titanium, uranium, identifying other potential metal effectors of not normative growth, and the potential of them acting in combination.

### 3.3. Developmental Milestones at 18 Months of the Cohort Born in 2015, Determination of the Metal Load in a Subgroup of Mother Toddlers Pairs, and Comparison to That Found at the Time of Delivery

The infants of the cohort born in 2015 once toddlers of 18 months underwent pediatric follow up. Of the original 502 mother–baby pairs seen at birth and 392 at 6 months, 308 pairs joined (62.3 of the initial T1 cohort). In Table 3, the results of the pediatric examination are reported.

The high percentage of below the norm (2SD) weight for age and length for age showed an even more unbalanced population than at 6 months, with a higher prevalence of underweight (33.1%) and stunting (33.9%); 26.4% of the toddlers were below average in weight/height standards, i.e., wasting; head circumference was also below the norm in 35.5% of the toddlers. Values above the norm were less frequent: 15.1% for weight and 7.7% for height. The prevalence of restricted development in the toddlers, especially that of underweight, was greater than at 6 months when the infants were milk fed. Muscle strength was mostly within the norm (83.3%).

Comparisons between mother-reported infant weight and height at 6 months and pediatric developmental indicators of toddlers at 18 months showed three statistically significant associations. Infants whose weight/age (Chi^2^ = 4.338, *P* = 0.037, N = 282) and height/age (Chi ^2^ = 15.338, *P* = 0.004, N = 282) were below average at 6 months had higher probabilities to be below average weight for age at 18 months. Furthermore, infants whose height was below average at 6 months had a higher probability to have below average head circumference for age at 18 months (Chi ^2^ = 16.541, *P* = 0.002, N = 282).

Among the 308 mother-toddler pairs, the hair collected from 80 pairs was randomly chosen for analysis of the content in metals of hair (Table 4); due to an insufficient amount for the lab in some of the samples, only 79 mothers’ and 69 toddlers’ hair gave reliable results. The toddlers were walking and had been exposed directly to environmental contamination through food and air and touching and the metal load in their hair was the result of the exposures they had in the last 4–5 months, as it was for that in their mothers’ hair.

The load of metals in the hair of the 79 mothers was found to be similar to the level they had at delivery, with the exception for a lower level of barium and a higher one for cadmium and titanium (Table 4, columns in A). The load in metals of the 69 toddlers was significantly higher for all metals than the level they had acquired in utero (Table 4, columns in B), consistent with their exposures to the environment, with the exception for molybdenum. For all metals, the contamination of the toddlers was lesser in amounts than that of their mothers, but their load in selenium and arsenic were higher, and molybdenum similar to that in the mothers.

### 3.4. Association between the Load of Metals Measured in Mothers and Progeny at Delivery/Birth and the Developmental Milestones of Toddlers at 18 Months

One aim of our study was to analyze whether the load of metal in mothers at the time of delivery, as well as that of newborns, could influence the later physical development of the infant, classified according to WHO standards. The highest barium in a mother’s hair was associated to toddlers below weight/age as well as the highest maternal molybdenum with lowest muscle strength in toddlers. The highest molybdenum in newborns was associated with lower weight/age, and the highest titanium was associated with the lowest weight/length (not shown). When similar analysis was conducted for the association the of the load of metals measured in mothers and infants at 18 months (T3) with the pediatric outcomes of toddlers, no significant association was found for any metal (data not shown).

## 4. Discussion

The prevalence of birth-defect babies has increased in Gaza since the start of aerial attacks following the removal of the Israeli occupation army and their settlers in 2005 [17]; health at birth continued to decrease in the aftermath of the three major military aggressions (2008–2009, 2012 and 2014), registering an increase in preterm births and birth defects associated to heavy metal contamination of pregnant women [4,8] and specific contamination in utero of newborn was documented [4,55]. These data, and the possibility that in utero metal contamination could affect the further development of the infant—as from reports of negative effects of metal assumption on development and neurocognitive functions [19,20,21,22,23,24,25,26,27,28,29,30,31,32,33,34,35,36]—according to the DOD (developmental origin of diseases) concept [2], led to the design of the present investigation.

Here, we show that newborns of Gaza have assumed relevant amounts of metals in utero all along the postwar era, from 2011 to 2019. The level of contamination in newborns could not be compared with standards from not war areas for lack of appropriate references from areas not heavily contaminated by industry, mining or water issues, or by wars. Yet, the level of metal contamination in the hair of cohorts of mothers delivering in the years 2015, 2016, 2018–2019 was previously compared with that of available adult standards from areas not at war [59], and 10 of these metals—with the exception of selenium and molybdenum—were found at each time point tested to be in higher concentration in Gaza women than in the references. This shows the stability of metals in the environment and illustrates their widespread diffusion after the attacks. Our documentation of chronic contamination by metals of the newborns in utero in the years 2015, 2016, 2018–19 is coherent with what found in their mothers [4,8]. The amounts of each metal found in the hair of newborns was in no case higher than in their mothers, except for molybdenum in 2015; in particular, here shown for the year 2015, the load in newborns for Ba, Sr and U was at least an order of magnitude lower, was lower for V, while it was similar to that of their mothers for As, Cd, Co and Ti. Differential trans-placental passage for different metals has been reported before [8,16].

The trend of change in time in the load of metals in newborns showed that the kind of contaminants of newborns in 2011 differed from that prevalent in the years following the 2014 attacks, remaining similar in kind and amounts thereafter; e.g., barium, arsenic, cadmium and uranium were highest in 2011 and remained at a stable level from 2015 onwards. These differences are likely due to the difference in weaponry known to be prevalent in the attacks in 2009 and 2014 [8]. In time, arsenic and strontium decreased and mercury (not significant) and vanadium increased slightly.

The trend in time of the load of individual metals in the newborns corresponds to the trend previously reported [6,8] in their mothers for Ba, Mo, U and V and is compatible with it for all other metals, with the exception of mercury and strontium, metals for which there was an opposite trend for mothers and newborns. We have no rationale to offer that explains this last observation.

At 6 months, the infants of this cohort were 20% underweight and 30% stunting, with no difference between sexes. In a retrospective study, it was reported [60] that underweight infants born in the year 2012 in Gaza had a prevalence of, respectively, 5% of underweight and 9% of stunting, suggesting a significant increase in these groups by the year 2015 and after the attacks in 2014.

The loads of metals at the time of delivery in mothers and newborns were associated with diverse outcomes in terms of the weight/age and/or length/age of the infants when 6 months old. The main sources of environmental metal contamination for the infants at 6 months of age were mostly limited to milk, water and breathing; in these circumstances, the contamination in utero may be a relevant factor affecting their development. An inverse relationship was found between higher load in mothers or newborns of some metals and lower height/age, weight/age, or both, suggesting that a higher load of these metals impaired the potential of growth of the 6-month-old infants. The role of high arsenic in the mother hair and of barium in the newborn on later growth are consistent with other studies [6,8,49,61].

Although underweight and stunting at 6 months could be influenced by low weight at birth, in this cohort, newborns of less than 2.5 kg were only 3.5%, a small fraction compared to the underweight infants. There are, nonetheless, uncertainty in interpreting the association of metal content with the cases with weight or length above the normative measures for their age; these are exemplified, e.g., by the fact that arsenic at its highest level in mother was associated with below average weight/age of the baby, but at its highest level in the newborn was associated with above average weight of the infant. Other metals in high load were associated with not normative growth—both below and above normal—e.g., higher chrome in mother and newborn with length/age of the infant both below and above the norm. A reason for these incongruences in the results of statistical analysis for infants above normative parameters for growth is that the numbers in the categories were small, yielding less reliable statistic values for significance. We have not investigated if the likely chronic contamination in infants through the milk of mothers, themselves chronically exposed to metals, might also interfere with the growth of the suckling infants (90% of them breast fed, although only 18% exclusively breast fed). All infants had more than one metal in their hair at birth and it is possible that multiple types of metal contamination, even at a low level, may be relevant in affecting later growth; we did not have a large enough sample to do statistical analysis for simultaneous contamination by multiple metals, an approach that could reveal eventual synergic or antagonistic effects of different metals, and threshold concentration for effectiveness of each metal in inducing metabolic and epigenetic changes in the mother, in the fetus and the first months of life of infants. Still, regardless of the complex scenario, at 6 months of age, infant development was restricted by in utero exposure to high loads at least of arsenic and barium. Both metals were implied in restriction of growth in utero, and barium in preterm birth [55].

In time, the growth impairment in the cohort studied was progressive, and the prevalence of underweight and of stunting became higher compared to that in six-month-old babies when the infants reached 18 months of age: 31% of the toddlers were underweight versus 20% at 6 months, and stunting was 33.9% at 18 months versus 30% at 6 months. In addition, at 18 months, 26.4% of the toddlers were wasting.

When compared with data reported in previous studies, it emerges that the achievement of milestones of growth was impaired progressively in the years in Gaza since the second intifada (started in the year 2000) and the serious restrictions of goods imposed by the occupation by Israel. The prevalence of stunting was reported for preschoolers in various retrospective studies: in 2003 as below 12% [62], 7.2% in 1996 and 10.9% in 2010 [63,64], 15% in 2009 and 19.6% in 2013 [65]; in a study focused on 6- and 24-month-old infants in Gaza, stunting was reported at 20% in 2014 [66]. In 2002, 17.5% of children, 12–23 months old, showed chronic malnutrition, 13.2% were underweight in 2006, and wasting was 12.7% in 2013. We show here that, in 2016, prevalence for underweight and stunting was higher than was previously reported.

The environment remained highly contaminated by metal war remnants in Gaza, and the same women tested in 2015 continued to assume not distinguishable levels of all metals in 2016, except for titanium and barium. The 2016 metal loads of women thus confirms in a specific cohort of the same people what was reported before analyzing different cohorts along the years [6,8].

In the hair of toddlers at 18 months, all environmental metals, except molybdenum, had loads exceeding those found when the same individuals were babies just born, showing that infants, once directly exposed to the environment through solid food and skin contact acquired higher amounts of metals in their hair compared to the level they had while in utero.

Metal levels in toddlers were lower, except for molybdenum, than that of their mothers measured at the same time. The observation is compatible with the reports that children accumulate lower levels of metals in hair from the environment than their adult kin, possibly due to different metabolism, difference in foods and activities between adults and infants.

Investigation of the association of the not normative growth with the load of metals that the toddlers had when they were born, or with that of their mothers at delivery, showed that the only significant association of below standard growth in toddlers was with a high level of metals different than those associated to the impairment in their growth parameters at 6 months, suggesting that to retrace univocally the effects of in utero exposure in a context where continual exposure to the contaminants occurs and the growth of the infant to toddler is additionally subject to many other potential stressors of normal development is critically difficult. Molybdenum measured in mothers and toddlers in 2016 and in mother at birth have values not exceeding that in not war areas, while its concentration in newborns was significantly higher. In toddlers, the correlation of higher molybdenum when they were newborns with underweight at 18 months suggests that it is of relevance for later growth. Notwithstanding this, the context in Gaza made it difficult to reach full certitude about the associations between toddlers’ growth and their exposure in utero.

When we planned this work, we were unaware of the persistence to be found, at almost unabated level, of metal war remnants in the environment of Gaza, of the extreme difficulties the country would have to face in order to remove the debris and of the length of time these would stay in open air, but also of the severe food and employment crisis that followed the attacks in 2014 an continues for years since [66]. These factors were consequences of the blockade and the lack of mechanical and economic means for environmental remediation and to restart local production, imposed by increasing more severe conditions in the aftermath of the attacks. By 18 months of age of the cohort born in 2015, other factors may have become of dominant relevance for the further impairment of the development of the infant and obscure the eventual role of fetal exposure and the eventual relevance of the progressive acquisition of metals in the infant’s body. This is also suggested by the fact that the relevance of birth weight on the outcome of the pediatric standard evaluation (analysis of variance) showed only one association: high birth weight predicted higher length/age at 18 months (F (2.277) = 4.372, *p* = 0.014, but birthweight was not significantly associated with weight/age, stunting, head circumference or muscle strength (not shown); in this cohort, the prevalence of babies of less than 2.5 kg at birth was 3.5% and that of babies over 4.5 kg was 1.7%. Chronic food scarcity is known to negatively affect infant development [63,64]. We previously documented that low animal protein intake of mothers was associated with an increased prevalence of babies of low weight born in the year 2016 [6], signaling that food unbalance and scarcity were diffuse at the same time when the toddlers in this cohort started a solid diet.

## 5. Highlights and Conclusions

We report persisting fetal contamination by metals war remnants over the years covering the aftermath of the three major military attacks in Gaza, from 2011 to 2019.

We found that, in infants that were in the womb during the attacks in 2014 at 6 months, there was an inverse correlation of underweight with metal contamination measured at birth, specifically with the level of arsenic in mothers and of barium in newborns.

The prevalence of underweight and stunting of the 6 month old in this cohort was more pronounced than that reported by other authors in previous years and increased in this group of infants that we followed longitudinally up to 18 months of age, when wasting also becomes highly frequent.

We measured the ongoing (chronic) exposure of women and their progeny during the 18 months of follow up in this study: metal contaminants in toddlers’ hair reached levels much higher than those that some these individuals had when measured at birth, while mothers’ loads in metals were similarly high at T1 and at T3, 18 months later.

Chronic assumption of metals along the 18 months may be one of the reasons why the correlation, still present for infants at 6 months, between the weight of the infant and a mother’s high level of arsenic at delivery or newborn high barium was no more found later. A firm conclusion about the relevance of the in utero exposure on toddler’s measurable growth parameters is thus hindered.

The chronic intake of multiple heavy metals during infancy may be a relevant cofactor of poor growth of which we could not test the relevance. Possibly multifactorial determinants are at play to influence growth of the toddlers, among these the increasingly poorer alimentation after the 2014 war due to the increased duress of the siege.

The *main limitation* of the study is that, when designing a longitudinal study, we could not have anticipate that the environmental sources of contamination would persist all along the time of the study, and socioeconomic and food availability, also factors of relevance on our pediatric measurables, would worsen severely, making it very difficult to identify the effect of the initial in utero exposure of the baby in toddlers.

The number of samples was not high enough to grant an analysis for the potential effect of simultaneous contamination by multiple metals. This kind of analysis could reveal eventual synergic or antagonistic effects of different metals, and indicate threshold concentration for the effectiveness of each metal in inducing metabolic and epigenetic changes. Given the complex pattern of contaminants in compresence, this kind of study potentially very informative, would require the analysis a much higher number of samples to achieve significance.

The *main novelty* of the study is that it is a prospective longitudinal study, allowing one to document in a cohort the chronicity of contamination of the women as well as the intake of metals since the in utero state and until infants are 18 months old; it allows one to identify the association of in utero contaminants with the growth of infants at 6 months and, overall, to show the negative impact of the aftermath of war on the health safety of infants. This report knits together the longitudinal approach and that of analysis at successive points in time in similar groups and confirms the consistence of the results and the complementarity of these approaches for assessment of contamination.

It points to the responsibility of the sieging countries Israel since 2007 and Egypt since early 2014 in having made impossible for years to remediate the environment in Gaza and thus reduce the risks for the health of the population, particularly, but not only, the youngest.

## Figures and Tables

**Table 1:** Comparison of metal concentration in hair of newborns at different time points (2011, 2015, 2016, and 2018–2019): medians and interquartile ranges.

	2011 (N = 69)		2015 (N = 503)		2016 (N = 50)		2018-19 (N = 18)		
Metal	Median	IQR	Median	IQR	Median	IQR	Median	IQR	*Test-Value*
Barium (Ba)	0.722 ^a^	0.743	0.460 ^b^	0.575	0.475 ^ab^	2.225	0.380 ^ab^	1.125	0.0001
Arsenic (As)	0.062 ^a^	0.108	0.015 ^b^	0.014	0.016 ^b^	0.024	0.010 ^c^	0.003	0.0001
Cadmium (Cd)	0.036 ^a^	0.055	0.009 ^c^	0.016	0.014 ^c^	0.046	0.007 ^c^	0.009	0.0001
Cobalt (Co)	0.053 ^a^	0.081	0.060 ^b^	0.460	0.030 ^c^	0.033	0.150 ^d^	0.128	0.0001
Chrome (Cr)	0.461 ^a^	0.483	0.180 ^b^	0.320	0.425 ^ab^	0.727	0.190 ^b^	0.128	0.0001
Mercury (Hg)	0.054	0.173	0.058	0.089	0.054	0.115	0.070	0.094	0.087
Molybdenum (Mo)	0.000 ^a^	0.000	0.214 ^b^	0.129	0.093 ^c^	0.074	0.199 ^b^	0.101	0.0001
Selenium (Se)	0.257 ^a^	0.310	1.010 ^b^	0.480	0.465 ^c^	0.208	1.690 ^d^	1.280	0.0001
Strontium (Sr)	na.	na.	3.000 ^b^	2.040	2.060 ^c^	6.435	2.215 ^c^	2.005	0.013
Titanium (TI)	0.000 ^a^	0.000	0.295 ^b^	0.490	0.140 ^b^	0.610	0.430 ^b^	0.525	0.0001
Uranium (U)	0.010 ^a^	0.023	0.003 ^b^	0.004	0.005 ^ab^	0.061	0.004 ^ab^	0.006	0.0001
Vanadium (V)	0.010 ^a^	0.010	0.014 ^b^	0.024	0.012 ^ab^	0.402	0.021 ^b^	0.022	0.0001

Legend IQR = Interquartile Range; *p*-values by Kruskal–Wallis tests with pairwise comparisons between samples from four different time periods; na = not available values/information. The significance values were adjusted by the Bonferroni corrections for multiple tests. For Pairwise Comparisons post-hoc statistics rows-wise, the different superscript (^a^, ^b^, ^c^, and ^d^) indicate statistical significance (p < 0.05) between the median values.

**Table 2:** Significant associations between heavy metal concentrations (ppm) in mother’s and newborn hair measured at birth and infant weight and height at 6 month (mother-reported): medians and interquartile ranges.

ppm	Barium (Ba)		Arsenic (As)		Cadmium (Cd)		Cobalt (Co)		Chrome (Cr)		Mercury (Hg)	
Mother Reported ^a^	Median	IQR	Median	IQR	Median	IQR	Median	IQR	Median	IQR	Median	IQR
	**Mother’s Metal Load in Hair in Delivery**											
Weight												
Below	3.710	6.300	0.085 ^a^	0.114	0.050	0.072	0.045	0.070	0.750	1.210	0.148	0.199
Average	4.985	7.590	0.062 ^b^	0.072	0.042	0.066	0.045	0.100	0.670	0.790	0.187	0.306
Above	8.620	12.52	0.028 ^c^	0.114	0.103	0.153	0.065	0.320	0.670	0.850	0.141	0.221
Kruskal-Wallis			10.590, *p* = 0.005									
Height												
Below	4.290	6.340	0.067	0.081	0.049	0.074	0.050 ^a^	0.011	0.810 ^a^	1.060	0.138	0.215
Average	4.595	5.920	0.065	0.078	0.038	0.062	0.040 ^a^	0.090	0.625 ^b^	0.750	0.188	0.276
Above	5.155	14.11	0.056	0.056	0.043	0.071	0.170 ^b^	0.210	1.065 ^c^	1.390	0.161	0.236
Kruskal-Wallis							8.562, *p* = 0.014		11.610, *p* = 0.003			
	**Newborn metal load in hair in birth**											
Weight												
Below	0.520 ^a^	0.780	0.015 ^a^	0.013	0.006	0.010	0.010	0.020	0.200	0.340	0.048	0.054
Average	0.405 ^b^	0.420	0.011 ^b^	0.011	0.006	0.014	0.010	0.000	0.120	0.190	0.052	0.078
Above	0.330 ^ca^	0.500	0.022 ^b^	0.018	0.007	0.013	0.010	0.060	0.640	1.980	0.059	0.076
Kruskal-Wallis	7.586, *p* = 0.023		15.412, *p* = 0.0001						11.412, *p* = 0.003			
Height												
Below	0.495 ^a^	0.570	0.015	0.130	0.005	0.009	0.010	0.010	0.180 ^a^	0.200	0.048	0.081
Average	0.370 ^b^	0.430	0.011	0.120	0.048	0.006	0.010	0.001	0.115 ^b^	0.200	0.052	0.091
Above	0.460 ^a^	0.380	0.024	0.073	0.037	0.009	0.010	0.010	0.190 ^a^	0.260	0.041	0.052
Kruskal-Wallis	11.253, *p* = 0.004		17.929, *p* = 0.0001						11.965, *p* = 0.003			
**ppm**	**Molybdenum (Mo)**		**Selenium (Se)**		**Strontium (Sr)**		**Titanium (TI)**		**Uranium (U)**		**Vanadium (V)**	
**Mother reported ^a^**	**Median**	**IQR**	**Median**	**IQR**	**Median**	**IQR**	**Median**	**IQR**	**Median**	**IQR**	**Median**	**IQR**
	**Mother’s Metal Load in Hair in Delivery**											
Weight												
Below	0.072	0.085	0.680	0.180	52.900	38.900	0.275	0.210	0.176	0.197	0.456	0.709
Average	0.059	0.061	0.660	0.200	48.700	47.930	0.245	0.260	0.157	0.195	0.442	0.539
Above	0.065	0.116	0.479	0.440	81.100	85.450	0.510	0.43	0.099	0.541	0.243	0.864
Height												
Below	0.070	0.090	0.065	0.020	58.500 ^a^	40.150	0.240	0.200	0.166	0.209	0.531 ^a^	0.635
Average	0.062	0.060	0.660	0.170	43.250 ^b^	39.250	0.250	0.290	0.167	0.199	0.389 ^b^	0.519
Above	0.076	0.073	0.655	0.180	79.450 ^c^	63.450	0.200	0.270	0.156	0.281	0.560 ^a^	0.737
					21.373, *p* = 0.0001						8.536, *p* = 0.014	
	**Newborn Metal Load in Hair in Birth**											
Weight												
Below	0.200	0.138	0.990	0.480	3.030 ^a^	1.960	0.290	0.320	0.002	0.002	0.013	0.018
Average	0.192	0.119	0.960	0.370	2.835 ^b^	1.748	0.240	0.330	0.001	0.003	0.011	0.015
Above	0.294	0.133	0.930	0.490	5.010 ^c^	3.785	0.400	0.320	0.003	0.008	0.024	0.024
Kruskal-Wallis					17.500, *p* = 0.002		6.904, *p* = 0.032					
Height												
Below	0.230	0.133	0.965	0.410	3.054	1.920	0.305 ^a^	0.450	0.002 ^a^	0.003	0.012	0.014
Average	0.178	0.114	0.950	0.380	2.776	1.845	0.210 ^b^	0.340	0.001 ^a^	0.003	0.011	0.018
Above	0.205	0.110	0.880	0.260	3.139	0.658	0.350 ^a^	0.810	0.029 ^b^	0.012	0.019	0.031
Kruskal-Wallis	18.147, *p* = 0.0001						25.830, *p* = 0.0001		12.374, *p* = 0.002			

Legend. Mother-reported weight and length of the infants of 6 month were classified according to WHO Child Growth Standards (2006). Kruskal–Wallis tests with Pairwise Comparisons between pediatric standard classifications. The Pairwise Comparisons post-hoc statistics column-wise, the different upper letters indicate statistical significance (*p* < 0.01) between the median values. The significance values adjusted by the Bonferroni corrections for multiple tests. The degrees of freedom varied for the weight (2381) and height (2337) due to missing values. Notes: IQR = Interquartile Range.

**Table 3:** Health information at pediatric check-up at 18 months.

Infant Developmental Problems	%	N
None	65.4	174
Feeding	15.8	42
Sleeping	14.3	38
Bonding	1.9	5
Other	2.6	7
Child health (yes answers)		
Non-communicable disease	3.9	12
Under regular medication	3.8	11
Infectious disease	3.3	10
Ear infections	4.0	12
Allergies	5.5	17
Asthma	4.9	15
Has had serious accident/ hospitalization	16.5	47
Vision problems	3.7	11
Hearing problems	3.7	11
Speech or language problems	2.0	6
**Weight for age ^a^**		
Below average	33.1	99
Average	51.8	155
Above average	15.1	45
**Height for age ^a^**		
Below average	33.9	101
Average	58.4	174
Above average	7.7	23
**Weight and height for age ^a^**		
Below average	26.4	79
Average	58.2	174
Above average	15.4	46
**Head circumference for age ^a^**		
Below average	35.5	106
Average	58.2	174
Above average	6.4	19
**Muscle development for age ^a^**		
Below average	14.0	42
Average	83.3	249
Above average	2.7	8

Legend. The toddlers were visited by 5 pediatricians each in the area of their residence when they reached 18–19 months of age. Participants’ numbers differed due to missing data (From *n* = 308 to *n* = 298) ^a^ Based on WHO Child Growth Standards (https://www.who.int/childgrowth/standards/en/).

**Table 4:** Comparison of metal concentration in the hair of mothers and infants assessed at delivery/birth and 18 months later: medians and interquartile range.

	A—Mothers (n = 79)					B—Progeny (n = 69)				
	At Delivery		18 Months Later			At Birth		At 18 Months of Age		
Metal ppm	Median	IQR	Median	IQR	Sign-tests	Median	IQR	Median	IQR	Sign-tests
Barium (Ba)	5.500	13.87	3.775	9.750	−2.92 **	0.485	0.490	0.970	0.820	4.00 ****
Arsenic (As)	0.077	0.102	0.071	0.111	**0.57**	0.014	0.008	0.146	0.140	7.46 ****
Cadmium (Cd)	0.046	0.065	0.072	0.092	2.03 *	0.006	0.015	0.102	0.100	7.22 ****
Cobalt (Co)	0.040	0.080	0.042	0.088	**0.72**	0.010	0.000	0.020	0.003	2.72 **
Chrome (Cr)	0.680	1.110	0.750	1.090	**0.90**	0.120	1.110	0.130	0.640	6.05 ****
Mercury (Hg)	0.159	0.492	0.183	0.226	**0.90**	0.057	0.082	0.142	0.190	4.09 ****
Molybdenum (Mo)	0.077	0.072	0.065	0.084	**1.70**	0.193	0.123	0.061	0.030	−7.71 ****
Selenium (Se)	0.655	0.190	0.715	0.480	**0.68**	1.020	0.320	1.080	0.320	2.72 **
Strontium (Sr)	56.500	52.930	37.650	44.470	**1.80**	3.030	1.615	5.020	6.630	3.85 ****
Titanium (TI)	0.260	0.230	4.21	6.040	8.78 ****	0.290	0.280	7.72	6.810	7.58 ****
Uranium (U)	0.168	0.222	0.111	0.143	**1.13**	0.002	00.002	0.060	0.060	7.64 ****
Vanadium (V)	0.537	0.523	0.604	0.545	**0.90**	0.013	0.013	0.323	0.360	7.56 ****

Legend. Median metal load in mother and progeny at delivery/birth and 18 months later. Sign-statistics tests the change in medians in the same population, here in the two assessment points. IQR = Interquartile Range. * *p* < 0.05, ** *p* < 0.01, **** *p* < 0.0001. Analytical data were for 79 mothers and 69 toddlers.

## Supplementary Materials

The following are available online at https://www.mdpi.com/1660-4601/17/18/6698/s1, Table S1: Demographic Background, Obstetric and Newborn Characteristics at birth (%).
