# Supplementary material for: It Takes Time to Unravel the Ecology of War in Gaza, Palestine: Long-Term Changes in Maternal, Newborn and Toddlers’ Heavy Metal Loads, and Infant and Toddler Developmental Milestones in the Aftermath of the 2014 Military Attacks
Source: Int J Environ Res Public Health. 2020 Sep 14;17(18):6698. doi: 10.3390/ijerph17186698 (PMC7558099; doi:10.3390/ijerph17186698)
Supplement: Supplementary file 1 [file ijerph-17-06698-s001.pdf]

Supplementary Table 1

Table S1. Demographic Background, Obstetric and Newborn Characteristics at birth (%).

	Participants[a]	
	**%**	***N***
Mother's age (years)		
16–20	14.5	73
21–30	62.9	316
31–40	21.1	106
41-52	1.4	7
Father's age (years)		
18–20	2.8	14
21–30	53.6	269
31–40	37.3	187
41-50	6.4	32
51-70	2.8	14
Number of children		
first child	26.5	133
1–3	53.0	268
4–6	17.0	85
7–10	3.5	18
Mother working status		
Works at home	88.8	446
Worker or entrepreneur	0.2	1
Blue collar: teacher, nurse	9.3	46
High professional	0.6	3
Student	1.2	6
Father working status		
No work	22.1	111
Worker	49.2	247
Entrepreneur	0.8	4
Blue collar: teacher, officer	22.5	113
High professional (doctor, engineer)	2.4	12
Student	0.8	4
Farmer	2.2	11
Type of residence		
Urban area	48.9	244
Village	18.6	93
Refugee camp	32.5	162
Parents' family relationship		
1st grade cousins	20.5	99
Relatives	14.1	68
Outside family	65.4	316
Child's sex		
Girl	50.4	253
Boy	49.6	249
Gestational age (weeks)		
< 37	4.2	20
37	11.3	54
38-42	84.5	404
Birth weight (gr)		
$\leq$ 2500	3.5	10
2510–3499	59.5	172
3500–4499	35.3	102
> 4500	1.7	5
Singleton-twin		
Singleton	98.6	495
Twins	1.2	6
Triples	0.2	1

Newborn health		
Excellent	51.1	406
Good	45.5	225
Reasonable	2.2	11
Severe health problems	0.4	2
Child still born	0.8	4
Birth defect		
ICD 10 diagnosis	5.2	26
Not defect	94.8	476
Type of delivery		
Vaginal	86.8	435
Caesarean	13.2	66

[a] Participant numbers differed due to missing data
